# Beyond Heart Failure: A Case of Missed Anti-neutrophil Cytoplasmic Antibody (ANCA)-Associated Glomerulonephritis

**DOI:** 10.7759/cureus.87973

**Published:** 2025-07-15

**Authors:** Ei Ei Phyu, Chesda Yit, Su Su San

**Affiliations:** 1 Internal Medicine, Nottingham University Hospitals NHS Trust, Nottingham, GBR; 2 Nephrology, Nottingham University Hospitals NHS Trust, Nottingham, GBR; 3 Internal Medicine, North Bristol NHS Trust, Bristol, GBR

**Keywords:** aki on ckd, antineutrophil cytoplasmic antibody (anca) associated vasculitis (aav), bnp, cyclophosphamide therapy, granulomatosis with polyangiitis (gpa), heart failure with preserved ejection fraction (hfpef), microscopic polyangiitis (mpa), mpo-anca, pauci-immune glomerulonephritis (gn), rituximab therapy

## Abstract

Anti-neutrophil cytoplasmic antibody-associated vasculitis, abbreviated as ANCA-associated vasculitis or AAV, presenting as pauci‑immune glomerulonephritis may be misdiagnosed in elderly individuals with comorbid cardiovascular disease.

We present a case of a 75-year-old female with type 2 diabetes mellitus, hypertension, atrial fibrillation and presumed heart failure with preserved ejection fraction (HFpEF) who developed progressive renal decline misattributed to diuretics and cardio-renal syndrome over eight months. Workup revealed myeloperoxidase anti-neutrophil cytoplasmic antibody (MPO-ANCA) positivity and renal biopsy confirmed pauci-immune focal proliferative glomerulonephritis. Induction therapy with high‑dose glucocorticoids and oral cyclophosphamide failed to avert dialysis dependency.

This case reflects that the interpretation of N-terminal pro-B-type natriuretic peptide (NT-pro BNP) in chronic kidney disease (CKD) is limited and overlapping symptoms complicate diagnosis. Current clinical guidelines recommend early ANCA testing, biopsy‑confirmed diagnosis, and induction therapy combining glucocorticoids with either rituximab or cyclophosphamide, with or without avacopan; plasma exchange may be considered for rapidly progressive glomerulonephritis. This case underscores the importance of early nephrology referral, ANCA serological screening, and prompt renal biopsy to avert irreversible renal injury despite guideline‑adherent therapy.

## Introduction

Anti‑neutrophil cytoplasmic antibody-associated vasculitis (ANCA-associated vasculitis or AAV) includes disorders such as granulomatosis with polyangiitis (GPA) and microscopic polyangiitis (MPA), frequently manifesting as rapidly progressive pauci‑immune glomerulonephritis and commonly presenting with nonspecific constitutional symptoms, pulmonary involvement, or upper airway disease, often mimicking other conditions such as infection or heart failure [1]. N-terminal pro-B-type natriuretic peptide (NT-pro BNP) is frequently used in clinical practice to support the diagnosis of heart failure, but its interpretation becomes challenging in the context of renal dysfunction, where elevated levels may reflect reduced clearance rather than true cardiac pathology [2]. Renal histopathological classification, as described by Berden et al., has prognostic implications. The extent of interstitial fibrosis and tubular atrophy (>40%) correlates with poor renal outcomes, even with optimal therapy [3]. The 2024 Kidney Disease: Improving Global Outcomes (KDIGO) and 2022 European League Against Rheumatism (EULAR) guidelines emphasize early diagnostic strategies including ANCA serology, renal biopsy, and induction therapy combining glucocorticoids with either rituximab or cyclophosphamide, with consideration of adjunctive avacopan and plasma exchange in severe cases [4-7]. Patients with multisystem comorbidities such as cardiovascular disease present diagnostic challenges that may delay intervention and reduce the likelihood of renal recovery.

## Case presentation

A 75‑year‑old female with a medical history of type 2 diabetes mellitus, hypertension, atrial fibrillation, presumed heart failure with preserved ejection fraction (HFpEF with left ventricular ejection fraction over 55% with diastolic dysfunction), and prior breast cancer experienced progressive renal deterioration over eight months, with a rapid decline in estimated glomerular filtration rate (eGFR) from 30 to 13 mL/min/1.73m². Symptoms including bilateral leg edema, shortness of breath, and lethargy were initially managed as heart failure with diuretics prescribed by her general practitioner, which provided only mild symptomatic relief. Home medications included ramipril, amitriptyline, bisoprolol, atorvastatin, furosemide, and lercanidipine. Over the last month, her renal function worsened (Table 1); urinalysis demonstrated proteinuria with a protein-to-creatinine ratio of 131 and hematuria, while urine cultures were negative. After stopping the ramipril and furosemide on admission, renal function failed to improve. 

**Table 1:** Progressive Decline in Renal Function and Hemoglobin Over Time and Noticeable Rapid Decline in Renal Function Over Last One-Month Period (February–March 2025) Hb - Hemoglobin, K - Potassium, eGFR - estimated glomerular filtration rate

	Mar 2025	Feb 2025	Nov 2024	Aug 2024	Apr 2024	Oct 2023
Hb	110 g/L	108 g/L	99 g/L	129 g/L	139 g/L	148 g/L
K	4.2 mmol/L	4.3 mmol/L	5.4 mmol/L	4.6 mmol/L	5.0 mmol/L	4.5 mmol/L
Urea	14.3 mmol/L	9.1 mmol/L	6.3 mmol/L	3.7 mmol/L	3.5 mmol/L	4.1 mmol/L
Creatinine	300 μmol/L	148 μmol/L	113 μmol/L	63 μmol/L	69 μmol/L	65 μmol/L
eGFR	13 ml/min/1.73m²	30 ml/min/1.73m²	41ml/min/1.73m²	84 ml/min/1.73m²	80 ml/min/1.73m²	81 ml/min/1.73m²
Albumin	33 g/L	34 g/L	29 g/L	30 g/L	33 g/L	37 g/L

On referral to nephrology, possible causes such as urinary tract infection, angiotensin-converting enzyme inhibitor-induced acute kidney injury, and diuretic effects were considered. B‑type natriuretic peptide (BNP) was markedly elevated above 11,000 ng/L. A comprehensive serological panel including myeloma screen, ANCA, anti-glomerular basement membrane (anti-GBM) antibodies, complement levels (C3, C4), and viral serologies showed high-titre myeloperoxidase (MPO)-ANCA positivity of 1280 CU (reference range: < 20 CU) while other test results were negative. 

**Figure 1:**
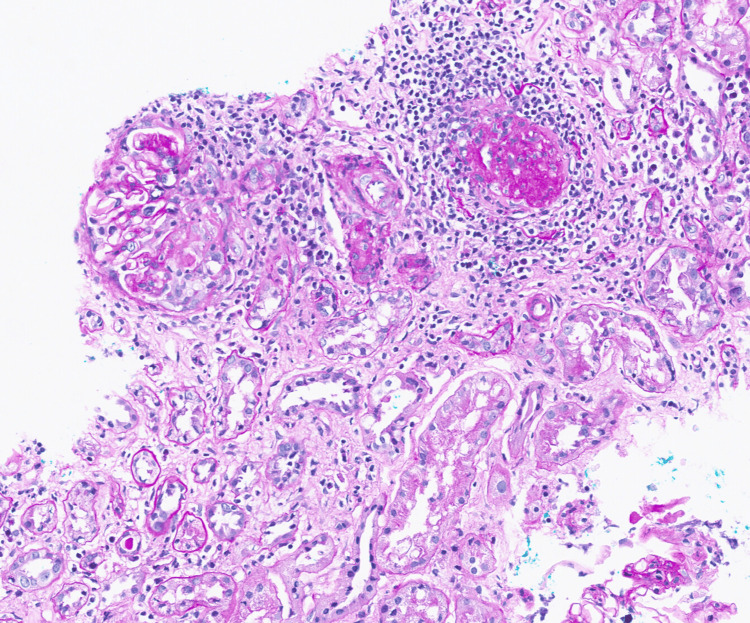
Periodic Acid-Schiff (PAS) stain demonstrating renal cortex with a glomerulus exhibiting a cellular crescent and another globally sclerosed glomerulus. Background shows interstitial inflammation and tubular atrophy.

**Figure 2:**
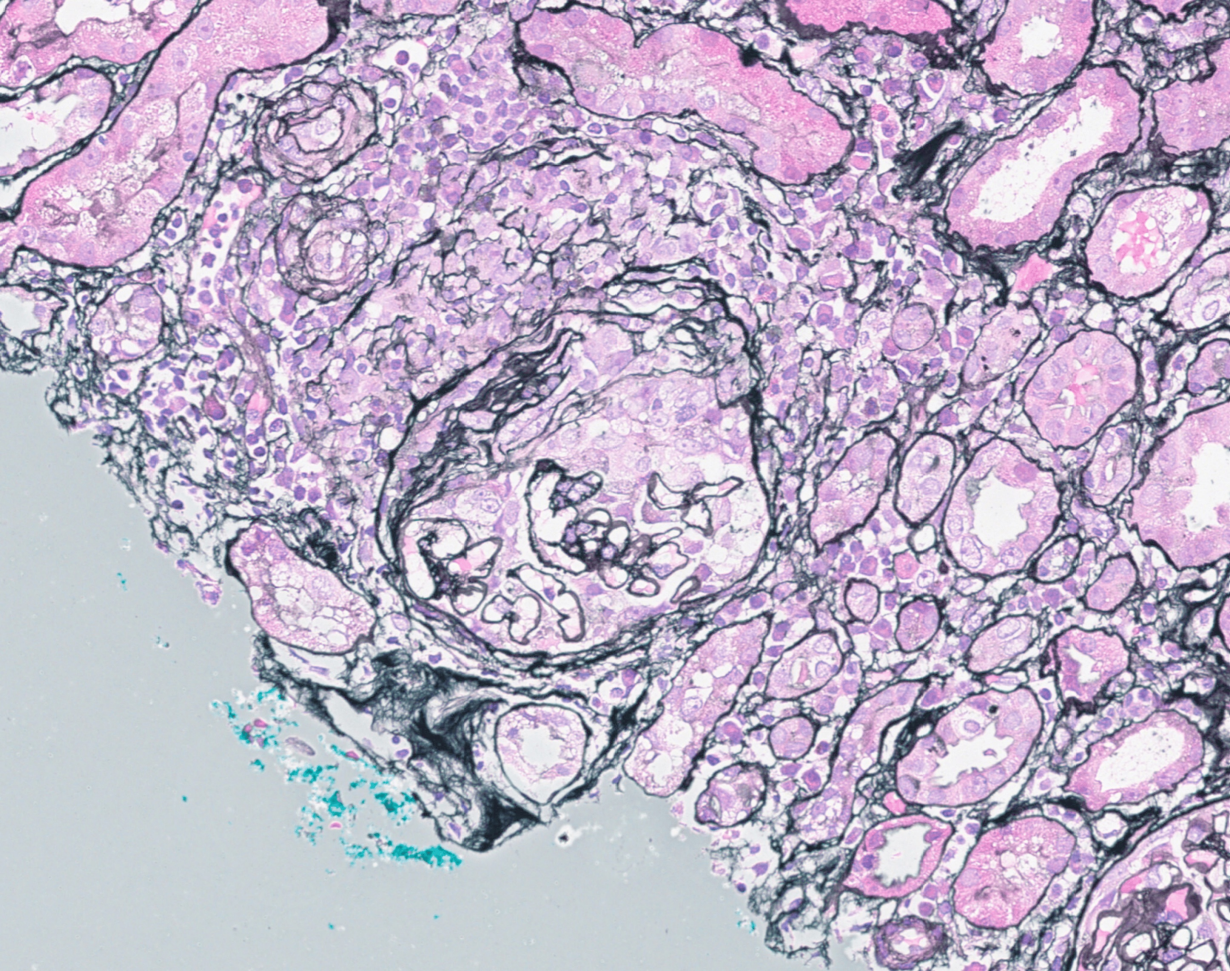
Silver stain of renal biopsy showing a glomerulus with a cellular crescent in Bowman’s space. The crescent is composed of proliferating parietal epithelial cells and infiltrating inflammatory cells. Surrounding tubulointerstitial compartment shows reactive changes.

**Figure 3:**
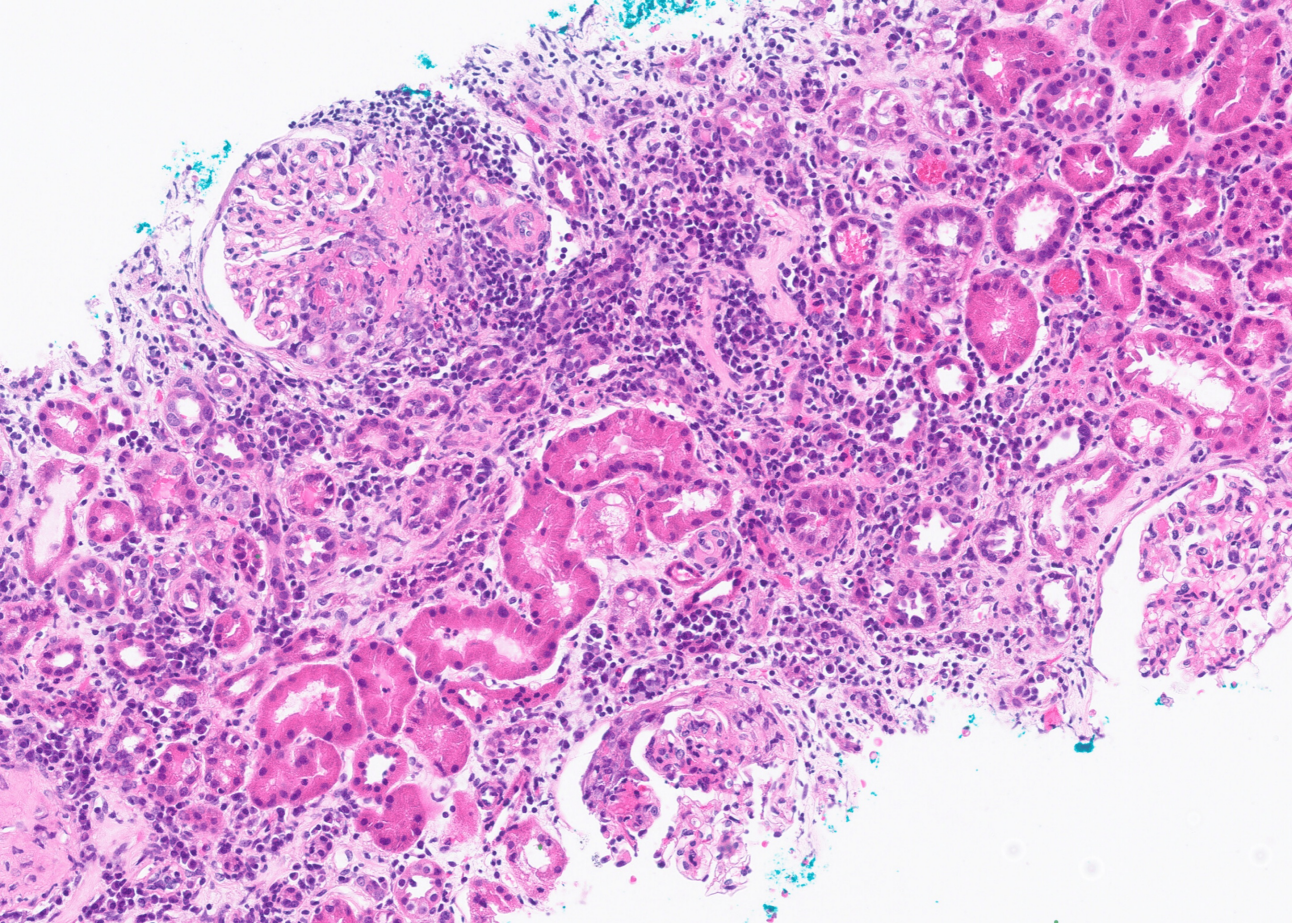
Hematoxylin and eosin (H&E) stained renal biopsy section. The glomeruli show segmental fibrinoid necrosis and crescent formation, characteristic of a necrotizing crescentic glomerulonephritis. Tubular lumens contain red blood cell (RBC) casts, and there is a prominent interstitial inflammatory infiltrate.

The subsequent native kidney biopsy showed features consistent with ANCA-associated focal proliferative glomerulonephritis. Histopathology report stated that of 18 glomeruli seen, four were globally sclerosed (Figure 1), one glomerulus had occlusive crescent (Figure 2), another glomerulus had segmental cellular crescent (Figure 3), and two glomeruli showed segmental fibrinoid necrosis (Figure 3). The remaining glomeruli were unremarkable. There was mild acute tubular injury with red cell casts (Figure 3) and moderate degree of interstitial fibrosis/tubular atrophy (IFTA) (45-50%) (Figure 2). The interstitium had moderate inflammatory infiltrate comprising lymphocytes, plasma cells, and eosinophils (Figures 1-3). The direct immunofluorescence was negative for immunoglobulins and complement components.

Induction therapy including oral prednisolone (following KDIGO tapering protocols targeting 5 mg/day by four to five months [7]) and oral cyclophosphamide was started following the diagnosis. Owing to irreversible biopsy changes, rituximab was not initiated. Despite prompt treatment, renal function failed to recover, and peritoneal dialysis was commenced by the 10th month.

## Discussion

ANCA-associated pauci-immune glomerulonephritis (AAGN) is a serious manifestation of small- to medium-sized vessel vasculitis that often presents with nonspecific systemic symptoms and progressive renal impairment [1,4]. This case illustrates several key diagnostic and therapeutic challenges encountered in the recognition and management of AAGN, particularly in elderly patients with overlapping comorbidities [4].

Diagnostic delay due to clinical overlap

Elevated B‑type natriuretic peptide is commonly used to support a diagnosis of heart failure, but its specificity is reduced in chronic kidney disease owing to impaired peptide clearance. In this case, persistently elevated levels misled the diagnostic process until further serological and histopathological investigation was undertaken - an approach now recommended by KDIGO, EULAR, and other major professional bodies [2-7].

Guideline‑based induction therapy

The 2021 American College of Rheumatology/Vasculitis Foundation guidelines (ACR/VF) and 2022 EULAR recommendations prioritize rituximab over cyclophosphamide for initial remission in severe renal presentations. Adjunctive avacopan may reduce corticosteroid exposure, and plasma exchange is advised for rapidly progressive glomerulonephritis [1,5-7]. The 2024 KDIGO update further reinforces early immunomodulatory therapy and rapid glucocorticoid tapering [7].

Histological predictors of outcome

Advanced chronic changes such as ≥ 40% interstitial fibrosis and tubular atrophy are strongly associated with poor renal prognosis even with optimal treatment [3]. Timely referral and biopsy may increase opportunities to deploy rituximab plus avacopan before irreversible damage occurs [7]. 

Limitations and lessons

Rituximab and avacopan were not used in this case due to the timing of presentation and the extent of chronic fibrosis. This underscores the need for clinicians to remain current with evolving guidelines and to act promptly when faced with suspicious clinical profiles [7].

## Conclusions

Clinicians should maintain a high index of suspicion for ANCA-associated vasculitis in elderly patients who present with unexplained renal impairment, even when heart failure-like symptoms are present. Elevated B‑type natriuretic peptide levels in patients with chronic kidney disease warrant careful interpretation, as these biomarkers can be misleading. Empirical serological testing for ANCA and early renal biopsy are essential steps in achieving a timely and accurate diagnosis. Once diagnosed, induction therapy aligned with current guidelines - utilizing rituximab or cyclophosphamide in combination with glucocorticoids, and possibly avacopan - should be started without delay. As demonstrated in this case, diagnostic or therapeutic delays can result in permanent kidney damage and the need for dialysis despite adherence to recommended treatment protocols.
